# White matter hyperintensities moderate neuropsychiatric response to N-acetylcysteine and exercise therapy in vascular mild cognitive impairment

**DOI:** 10.1177/13872877261490542

**Published:** 2026-10-07

**Authors:** Ethan Mah, Damien Gallagher, Yejin Kang, Fuqiang Gao, Joel Ramirez, Zahinoor Ismail, Sandra E. Black, Xingshan Cao, Nathan Herrmann, Susan Marzolini, Paul Oh, Jinghan Jenny Chen, Kate Survilla, Walter Swardfager, Danielle Vieira, Mark J. Rapoport, Krista L. Lanctôt

**Affiliations:** 1 Hurvitz Brain Sciences Program, 282299 Sunnybrook Research Institute, Toronto, ON, Canada; 2 Department of Pharmacology & Toxicology, 7938 University of Toronto, Toronto, ON, Canada; 3 Department of Psychiatry, 7938 University of Toronto, Toronto, ON, Canada; 4 Dr. Sandra Black Centre for Brain Resilience and Recovery, 282299 Sunnybrook Research Institute, Toronto, ON, Canada; 5 Graduate Department of Psychological Clinical Science, 33530 University of Toronto Scarborough, Toronto, ON, Canada; 6 Department of Psychiatry, Cumming School of Medicine, 70401 University of Calgary, Calgary, AB, Canada; 7 KITE Toronto Rehabilitation Institute, 7989 University Health Network, Toronto, ON, Canada

**Keywords:** Alzheimer's disease, clinical trials/studies, neuroimaging, neuropsychiatric symptoms, vascular cognitive impairment

## Abstract

**Background:**

Vascular mild cognitive impairment (vaMCI) is frequently accompanied by neuropsychiatric symptoms (NPS), particularly apathy, which are associated with white matter hyperintensities (WMH). N-acetylcysteine (NAC) combined with exercise may provide neuroprotective benefits.

**Objective:**

We hypothesized that baseline frontal and global WMH burden would moderate the neuropsychiatric response to NAC plus exercise. We further hypothesized that the strongest domain-specific effects would be observed for apathy and affective dysregulation.

**Methods:**

Participants with vaMCI were randomized to NAC (2400 mg daily) or placebo plus exercise for 6 months. WMH volumes were quantified using structural MRI. NPS were assessed using the Mild Behavioral Impairment Checklist (MBI-C) at baseline, 3 months, and 6 months. Negative binomial generalized linear mixed models examined WMH × time × treatment interactions. False discovery rate (FDR) correction was applied.

**Results:**

Fifty-one participants were included. Lower baseline frontal (*β* = 0.229, SE = 0.070, FDR-adjusted *p* = 0.007) and global (*β* = 0.258, SE = 0.080, FDR-adjusted *p* = 0.007) WMH volume was associated with greater reductions in NPS over 6 months in the NAC plus exercise group. This effect was driven by improvements in apathy (frontal: *β* = 0.186, SE = 0.076, FDR-adjusted *p* = 0.048; global: *β* = 0.207, SE = 0.089, FDR-adjusted *p* = 0.049).

**Conclusions:**

Baseline WMH burden moderates neuropsychiatric treatment response in vaMCI. These findings support the importance of optimizing care for cerebrovascular risk factors in this vulnerable population.

## Introduction

Vascular mild cognitive impairment (vaMCI) is an early-stage vascular cognitive impairment defined by cognitive deficits due to cerebrovascular disease that are not severe enough to be classified as dementia.^1^ In addition to cognitive deficits, individuals with vaMCI frequently exhibit neuropsychiatric symptoms (NPS), including apathy and mood disturbances.^2–4^ These NPS are clinically significant, as they can negatively affect daily functioning and health behaviors^5,6^ and increase the risk of progression to dementia among affected individuals.^7^

White matter hyperintensities (WMHs) are detectable as bright spots or confluent patches on T2-weighted and fluid-attenuated inversion recovery (FLAIR) magnetic resonance imaging (MRI) scans. WMHs are considered as markers of cerebral small vessel disease (CSVD) and commonly observed in vaMCI not due to strategic territorial infarcts or multiple lacunar infarcts. WMHs can reflect damage to white matter tracts connecting the frontal cortex with subcortical structures, notably disrupting the fronto-subcortical circuits crucial for motivation, goal-directed behavior, and emotional regulation.^8^ Evidence suggests that higher WMH burden undermines these circuits and exacerbates NPS.^3,8^ In a sample of memory clinic patients with MCI, mild behavioral impairment was associated with 9.4% greater WMH volume, demonstrating the link between NPS and WMH.^9^

The severity of WMH burden, particularly in frontal regions, has been linked to increased apathy and mood disturbances in vascular cognitive impairment.^4,10^ Apathy is characterized by symptoms of diminished initiative, decreased interest, and/or impaired emotional expression/responsiveness.^11^ Apathy has emerged as one of the most prominent NPS in vaMCI, with studies demonstrating its association with accelerated cognitive decline and increased dementia risk.^3,8,12^ Apathy is also associated with poorer self-care and reduced adherence to medical regimens.^4–6,13^ CSVD can be a primary cause of apathy, even in the absence of other vascular symptoms.^14,15^ Recent evidence suggests a causal relationship between CSVD and apathy, but evidence for a temporal relationship remains limited.^14^ Mood disturbances, including depression and anxiety, are also closely associated with WMH burden.^4,16–18^ This disruption can manifest as persistent low mood, loss of interest and pleasure, feelings of guilt or hopelessness, and increased emotional reactivity.^19^ Apathy and mood disturbances represent clinically important neuropsychiatric symptoms that may be related to WMH in vaMCI, underscoring the importance of understanding vascular contributions to behavioral health in aging.

WMHs are thought to arise from chronic cerebral hypoperfusion, which leads to collagenosis of the deep medullary venular system with leakage (vasogenic edema) evident on diffusion weighted MRI which further leads to cascading ischemic and inflammatory processes that result in elevated oxidative stress levels.^20–23^ Additionally, oxidative glial cell damage plays a key role in the pathological development of WMHs.^24^ Given these mechanisms, N-acetylcysteine (NAC), a potent antioxidant that serves as a precursor to glutathione (GSH), the brain's primary antioxidant, has been explored as a potential therapeutic agent to reduce oxidative stress in neurodegenerative conditions.^25^ Combined with structured aerobic and resistance training, which has significant positive effects on ameliorating NPS such as apathy, depression, and anxiety,^26,27^ NAC may offer synergistic effects for mitigating NPS in vaMCI.

This study was a secondary analysis of the MOVE-IT randomized clinical trial. MOVE-IT compared NAC supplementation and exercise training to placebo and exercise for improving cognitive outcomes in patients with vaMCI. This secondary analysis specifically examined whether baseline frontal/global WMH burden moderated NPS trajectories over six months. We hypothesized that lower baseline frontal and global WMH burden would moderate the neuropsychiatric response to NAC plus exercise, such that participants with lower WMH burden would experience greater reductions in NPS, as measured by the Mild Behavioral Impairment Checklist (MBI-C),^28^ compared with those receiving placebo plus exercise. Based on previous literature linking WMH burden to motivational and affective symptoms,^9^ we further hypothesized that the strongest domain-specific effects would be observed for apathy and affective dysregulation.

## Methods

### Interventions

MOVE-IT was a 6-month, randomized, double-blind, placebo-controlled, parallel-group trial designed to assess the safety and efficacy of oral NAC for improving cognitive outcomes in individuals with vaMCI participating in cardiac rehabilitation (CR).^25^ Participants were randomized to receive either 2400 mg of NAC plus exercise training or a matching placebo plus exercise training. All participants engaged in a comprehensive CR program at the University Health Network – Toronto Rehabilitation Institute (UHN-TRI), which incorporated aerobic and resistance training alongside risk factor management. Depending on their program length (16 or 24 weeks), participants attended weekly supervised sessions combining standardized aerobic exercise and resistance training with education sessions.^29–31^ In addition, they were instructed to complete home-based training consisting of four aerobic exercise sessions and one to two resistance training sessions per week. All study procedures were approved by the Research Ethics Boards (REB) of Sunnybrook Health Sciences Centre and UHN.

### Participant eligibility

MOVE-IT participants were adults aged 55–85 years who were proficient in English. Eligibility required evidence of stable coronary artery disease (CAD), defined as a prior myocardial infarction, significant stenosis in at least one major coronary artery, a history of revascularization, or the presence of two or more vascular risk factors such as hypertension, dyslipidemia, diabetes, physical inactivity, smoking, obesity, or sleep apnea. All participants also met diagnostic criteria for vascular cognitive impairment.^32,33^ Exclusion criteria encompassed prior stroke, epilepsy, uncontrolled or severe medical illnesses (including advanced diabetes or asthma), major psychiatric disorders (e.g., schizophrenia or bipolar disorder), and any form of dementia or other neurodegenerative disease. Individuals were further excluded if they had contraindications to MRI or NAC treatment, used daily nitroglycerin, or had surgery scheduled within 30 days. For inclusion in the present secondary analysis, participants additionally required baseline MRI and Center for Epidemiologic Studies Depression Scale (CES-D) data.

### Cognitive screening

All consenting participants completed the Montreal Cognitive Assessment (MoCA) to screen global cognitive status. Cognitive performance was then characterized in greater detail using the 60-min neuropsychological battery developed by the National Institute of Neurological Disorders and Stroke and the Canadian Stroke Network (NINDS-CSN),^34^ which evaluates executive function, verbal memory, visuospatial memory, working memory, and processing speed.^35^ Eligibility required a MoCA score below 28^36^ as well as performance of at least one standard deviation below published normative values in at least one cognitive domain.^37^ A threshold of 28, rather than the conventional 26, was applied to better reflect the high educational attainment of the UHN-TRI cohort.^29–31^

### Neuroimaging protocol

Structural MRI was obtained using a research-dedicated 3T Siemens Prisma scanner (Siemens Healthineers, Erlangen, Germany) in accordance with the Canadian Dementia Imaging Protocol.^38^ Image evaluation and ratings were conducted by a single radiologist blinded to participants’ clinical data. “Probable” vaMCI was defined by cognitive deficits (as previously described) in combination with diffuse, subcortical cerebrovascular pathology, indicated by either (1) at least two silent supratentorial infarcts and/or (2) significant white matter disease, reflected by an Age-Related White Matter Changes (ARWMC) scale score ≥2.^39^ Participants with cognitive deficits on the NINDS-CSN 60-min battery, clinical features consistent with a vascular origin (i.e., impairment in the presence of substantial vascular risk factors), and focal white matter lesions (ARWMC score = 1) were classified as “possible vaMCI,” reflecting a higher likelihood of vascular contributions to their impairments.

After tissue segmentation, 26 bilateral brain regions were parcellated using Semiautomatic Brain Region Extraction.^40^ WMH were identified on either FLAIR or PD/T2-weighted sequences using one of two semiautomatic approaches: the Fuzzy Lesion Extractor (FLEX)^41^ or Lesion Explorer.^42^ Trained neuroimaging analysts, blinded to all clinical information, performed quality control following standardized procedures to ensure accurate segmentation. The final outputs yielded volumetric estimates of periventricular and deep WMH (p/dWMH).

### Outcome measures

The MBI-C was administered to all participants (*n* = 51) at three time points during the trial: baseline, 3 months (midpoint), and 6 months (endpoint). At each assessment, a trained researcher administered the MBI-C directly to the participant using the same standardized instructions, ensuring consistent data collection across study visits. The MBI-C consists of 34 items grouped into five neuropsychiatric domains; decreased drive/motivation (apathy), affective dysregulation (mood/anxiety symptoms), impulse dyscontrol (agitation, impulsivity), social inappropriateness (impaired social cognition), and abnormal perception or thought content (psychotic symptoms), with each item rated for presence or absence of behavioral change.^43^ If a change was present, its severity was further rated on a scale from 1 to 3 (1 = mild, 2 = moderate, and 3 = severe).^43^ Responses are based on sustained changes in behavior lasting at least six months and represent a departure from the individual's longstanding patterns.^43^ Domain scores are calculated by summing endorsed responses within each domain, and a total MBI-C score is obtained by adding the individual domain scores, providing a quantitative measure of NPS burden.^43^ Although the instrument's criterion requires behavioral changes to persist for at least 6 months, the MBI-C was administered using the same standardized instructions and scoring procedures at baseline, 3 months, and 6 months. Repeated scores were used in this study to characterize longitudinal changes in neuropsychiatric symptom severity, and all three assessments were included in the mixed-effects models. The prespecified primary contrast, however, concerned differential change from baseline to the 6-month endpoint.

### Statistical analyses

To test the hypothesis that lower baseline frontal WMH burden would moderate the neuropsychiatric response to NAC plus exercise, such that participants with lower WMH burden would experience greater reductions in NPS compared with those receiving placebo plus exercise over the 6-month intervention period, we used negative binomial generalized linear mixed models. Covariates were age, sex, baseline MoCA, baseline CES-D scores (depressive symptoms), total number of vascular risk factors, and a measure of preserved brain parenchyma to account for brain atrophy. WMH volumes were log-transformed and adjusted for supratentorial total intracranial volume to account for individual differences in head size. Random intercepts were included to account for repeated measures and baseline differences in MBI-C scores between individuals. The frontal WMH models included a frontal brain parenchymal fraction proxy, calculated as the sum of normal-appearing grey matter and normal-appearing white matter within the frontal lobe divided by supratentorial total intracranial volume. Outcomes included the MBI-C total score (sum of domains 1–5) and the individual scores for domains 1–4. Individual domain 5 scores (psychotic symptoms) were excluded from analysis due to extremely low variance and zero-inflation, which caused model convergence issues. To avoid over-adjustment, CES-D scores were not included as a covariate in models where the MBI-C affective dysregulation domain was the outcome, as this domain substantially overlaps with depressive symptoms. These models were repeated using baseline global WMH volumes, with adjustment for global brain parenchymal fraction. To control for multiple comparisons, False Discovery Rate (FDR) correction was applied. The statistical program R (version 4.3.2) was used.

To assess the relationship between frontal and global WMH burden, a Spearman rank correlation was performed using baseline WMH measures. To evaluate the regional specificity of the observed associations, exploratory analyses were subsequently conducted using baseline occipital, parietal, and temporal WMH volumes. These models used identical covariates and interaction terms, with each model additionally adjusted for the corresponding regional brain parenchymal fraction proxy. False discovery rate correction was applied separately within each exploratory regional analysis.

## Results

### Demographics

Participant baseline demographics and physical/clinical characteristics are shown in Table 1. Participant baseline cognition and WMH volumetrics are shown in Table 2. There were no significant differences between baseline demographics or characteristics between the two treatment arms. There were also no significant differences in baseline MBI-C total and domain scores or WMH volumes.

**Table 1.** Baseline demographics and physical/clinical characteristics by treatment group.

	NAC (*n* = 24)	Placebo (*n* = 27)	Standardized effect size
Demographic Characteristics			
Age, years	66.9 (7.4)	68.9 (7.4)	−0.31 (95% CI: [−0.88, 0.26])
Male, *n* (%)	14 (58.3)	21 (77.8)	−0.42
Self-identified race/ethnicity, *n* (%)			
White	15 (62.5)	19 (70.4)	−0.17
Asian	4 (16.7)	3 (11.1)	0.16
South Asian	1 (4.2)	3 (11.1)	−0.27
Black	2 (8.3)	1 (3.7)	0.20
Another not listed above	2 (8.3)	1 (3.7)	0.20
Years of education	16.3 (2.4)	15.8 (3.5)	0.17 (95% CI: [−0.39, 0.74])
Full-time employed, *n* (%)	8 (33.3)	9 (33.3)	0.00
Physical/Clinical Characteristics			
BMI, kg/m^2^	26.8 (5.4)	27.4 (5.5)	−0.10 (95% CI: [−0.66, 0.46])
Systolic BP, mmHg	130 (15.60)	125 (16.80)	0.34 (95% CI: [−0.22, 0.92])
Diastolic BP, mmHg	79.9 (10.0)	78.3 (9.5)	0.17 (95% CI: [−0.40, 0.73])
Heart rate, b·min^−1^	64.9 (9.7)	64.6 (9.6)	0.04 (95% CI: −0.53, 0.60])
Vascular Risk Factors			
Hypertension, *n* (%)	13 (54.2)	16 (59.3)	−0.1
Diabetes, *n* (%)	4 (16.7)	8 (29.6)	−0.31
Dyslipidemia, *n* (%)	12 (50.0)	15 (55.6)	−0.11
Obesity, BMI ≥ 30, *n* (%)	4 (16.7)	5 (18.5)	−0.05
Smoking history (tobacco), *n* (%)	8 (33.3)	10 (37.0)	−0.08
Myocardial infarction, *n* (%)	6 (25.0)	9 (33.3)	−0.18
Number of vascular risk factors	1.96 (1.16)	2.33 (1.18)	−0.32 (95% CI: [−0.89, 0.25])

Values reported as count (percentage) or as mean (SD). Standardized effect sizes reported as Cohen's d for continuous variables and Cohen's h for proportions. NAC: N-acetylcysteine; SD: standard deviation; BMI: body mass index; BP: blood pressure.

**Table 2.** Baseline cognition and WMH volumetrics by treatment group.

	NAC (*n* = 24)	Placebo (*n* = 27)	Standardized effect size
NPS Burden and Cognition			
MBI-C total score	7.46 (9.18)	4.30 (3.74)	0.46 (95% CI: [−0.11, 1.03])
Apathy	2.46 (3.46)	1.41 (1.53)	0.40 (95% CI: [−0.17, 0.97])
Affective dysregulation	2.58 (4.07)	1.52 (1.76)	0.35 (95% CI: [−0.22, 0.91])
Impulse dyscontrol	2.00 (3.32)	1.26 (1.48)	0.29 (95% CI: [−0.27, 0.86])
Social inappropriateness	0.33 (0.82)	0.11 (0.32)	0.37 (95% CI: [−0.20, 0.93])
Abnormal perceptions or thought content	0.08 (0.28)	0.00 (0.00)	0.43 (95% CI: [−0.14, 1.00])
CES-D (total)	8.00 (6.08)	9.34 (6.87)	−0.21 (95% CI: [−0.77, 0.36])
MoCA (total)	22.90 (2.61)	23.10 (2.89)	−0.07 (95% CI: [−0.64, 0.49])
WMH Volumetrics (cm^3^ * 10^−3^)			
Frontal WMH	2.83 (2.82)	2.77 (5.12)	0.01 (95% CI: [−0.55, 0.58])
Frontal pWMH	2.52 (2.58)	2.41 (4.97)	0.03 (95% CI: [−0.53, 0.59])
Frontal dWMH	0.31 (0.57)	0.37 (0.66)	−0.09 (95% CI: [−0.65, 0.47])
Global WMH	7.42 (8.55)	7.65 (11.60)	−0.02 (95% CI: [−0.58, 0.54])
Global pWMH	6.61 (7.84)	6.96 (11.18)	−0.04 (95% CI: [−0.60, 0.53])
Global dWMH	0.81 (1.17)	0.69 (0.83)	0.12 (95% CI: [−0.44, 0.69])

Values reported as mean (SD). Standardized effect sizes reported as Cohen's d. NAC: N-acetylcysteine; MBI-C: Mild Behavioral Impairment Checklist; MoCA: Montreal Cognitive Assessment; SD: standard deviation; WMH: white matter hyperintensities; pWMH: periventricular white matter hyperintensities; dWMH: deep white matter hyperintensities.

### Frontal WMH

At study endpoint, there was a statistically significant three-way interaction among frontal WMH burden, time, and treatment group on overall NPS (MBI-C total score), after FDR correction (*β* = 0.229, SE = 0.070, FDR-adjusted *p* = 0.007) (Table 3). This indicates that participants with lower baseline frontal WMH burden experienced greater reductions in NPS over 6 months with NAC plus exercise compared to placebo plus exercise (Figure 1). To facilitate visualization of these findings, observed individual participant trajectories for MBI-C total scores are shown in Supplemental Figure 1.

**Figure 1.**
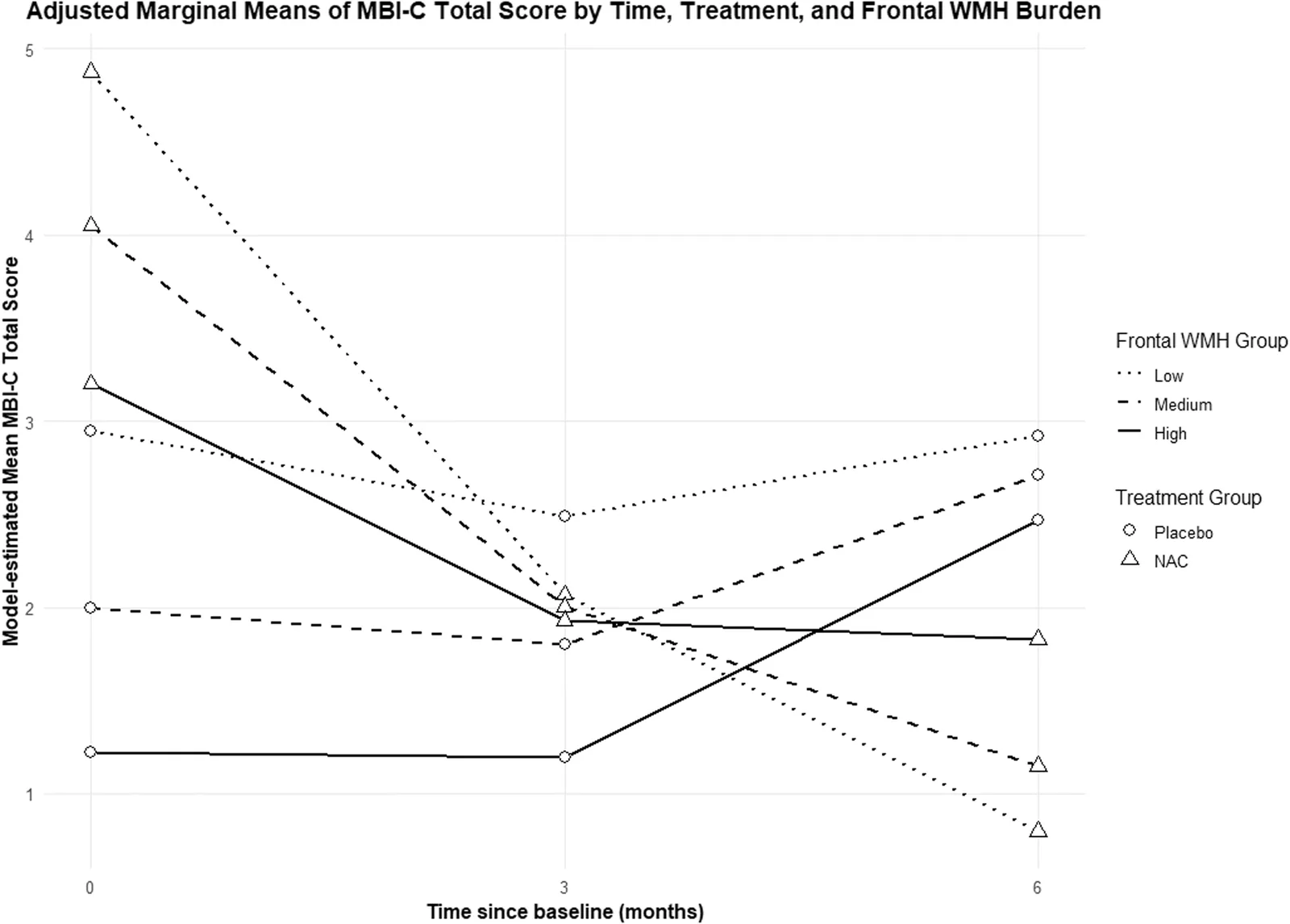
Adjusted marginal means of MBI-C total score over time by treatment assignment (NAC vs placebo) and frontal WMH tertile. Values represent model-estimated marginal means from the negative-binomial mixed model, adjusted for age, sex, baseline depression, baseline MoCA, vascular risk factors, and preserved brain parenchyma. WMH tertiles are shown for visualization only. Lower scores indicate fewer NPS. Line type denotes WMH tertile (dotted = low WMH; dashed = mid WMH; solid = high WMH).

**Table 3.** Frontal WMH × time × treatment interaction effects at endpoint on MBI-C total and domain scores.

Outcome	*β* (SE)	Nominal *p*	FDR-adjusted *p*	
MBI-C total score	0.229 (0.070)	0.001	0.007	**
Apathy	0.186 (0.076)	0.014	0.048	*
Affective dysregulation	0.126 (0.064)	0.049	0.090	.
Impulse dyscontrol	0.141 (0.091)	0.121	0.121	
Social inappropriateness	0.514 (0.267)	0.055	0.090	.

*p < 0.05; **p < 0.01; . *p* < 0.1. MBI-C: Mild Behavioral Impairment Checklist.

When examining MBI-C domains, the interaction effect was significant for the apathy domain (*β* = 0.186, SE = 0.076, FDR-adjusted *p* = 0.048), but not for affective dysregulation, impulse dyscontrol, or social inappropriateness after FDR correction (all FDR-adjusted *p* > 0.05) (Table 3). This indicates that participants with lower frontal WMH burden experienced greater reductions in apathy over 6 months with NAC plus exercise compared to placebo plus exercise (Figure 2). Observed individual participant trajectories for MBI-C apathy scores are shown in Supplemental Figure 2. The interaction was not significant for affective dysregulation (FDR-adjusted *p* = 0.090), impulse dyscontrol (FDR-adjusted *p* = 0.121), and social inappropriateness (FDR-adjusted *p* = 0.090), indicating possible trends which did not remain significant after multiple comparison adjustment.

**Figure 2.**
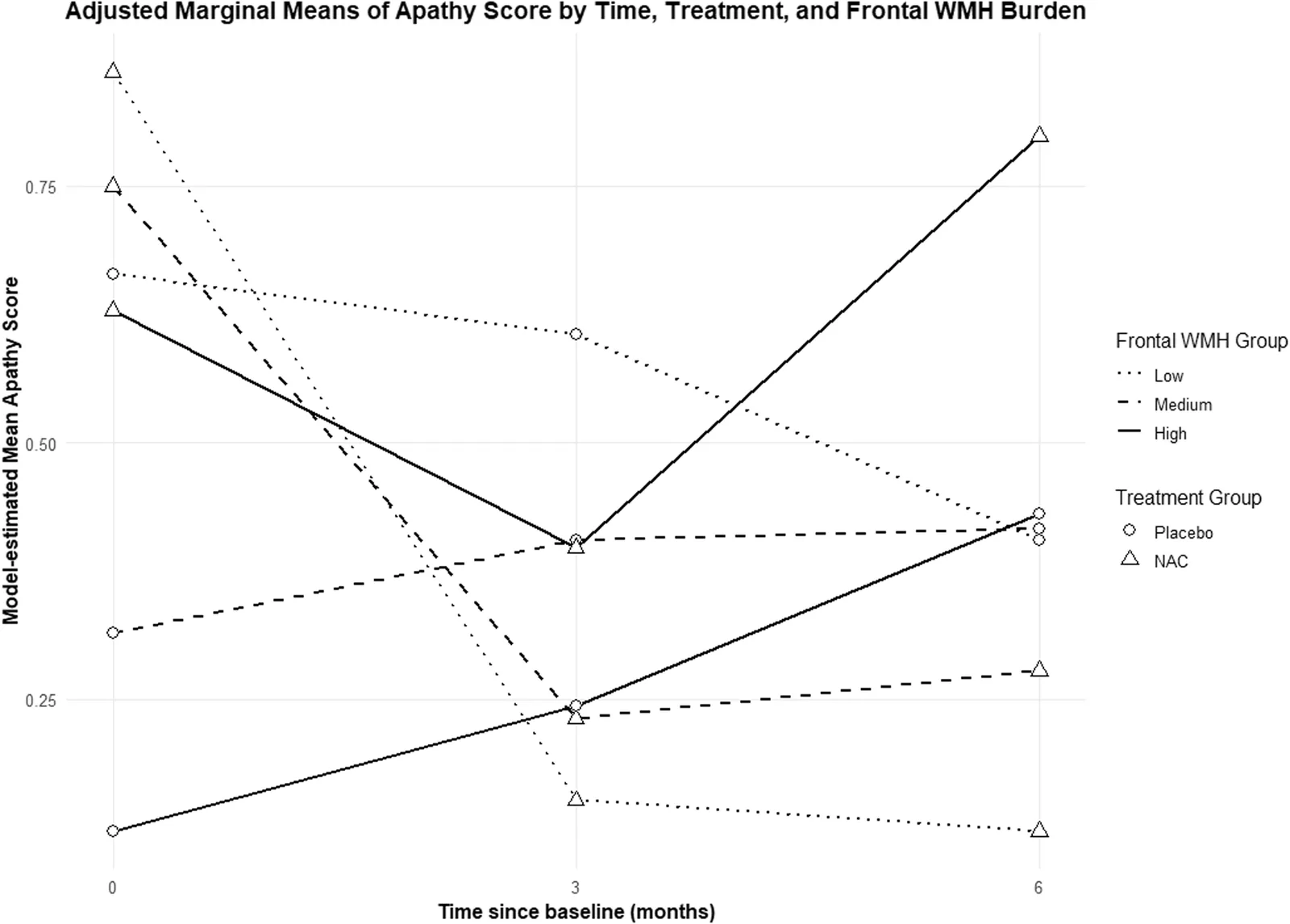
Adjusted marginal means of MBI-C apathy (domain 1) score over time by treatment assignment (NAC vs placebo) and frontal WMH tertile. Values represent model-estimated marginal means from the negative-binomial mixed model, adjusted for age, sex, baseline depression, baseline MoCA, vascular risk factors, and preserved brain parenchyma. WMH tertiles are shown for visualization only. Lower scores indicate fewer apathy symptoms. Line type denotes WMH tertile (dotted = low WMH; dashed = mid WMH; solid = high WMH).

### Global WMH

Similarly, at study endpoint, a significant three-way interaction was observed between global WMH burden, time, and treatment on overall NPS (MBI-C total score) after FDR correction (*β* = 0.258, SE = 0.080, FDR-adjusted *p* = 0.007) (Table 4). This indicates that participants with lower baseline global WMH burden experienced greater reductions in NPS over 6 months with NAC plus exercise compared to placebo plus exercise, consistent with the frontal WMH findings (Figure 3).

**Figure 3.**
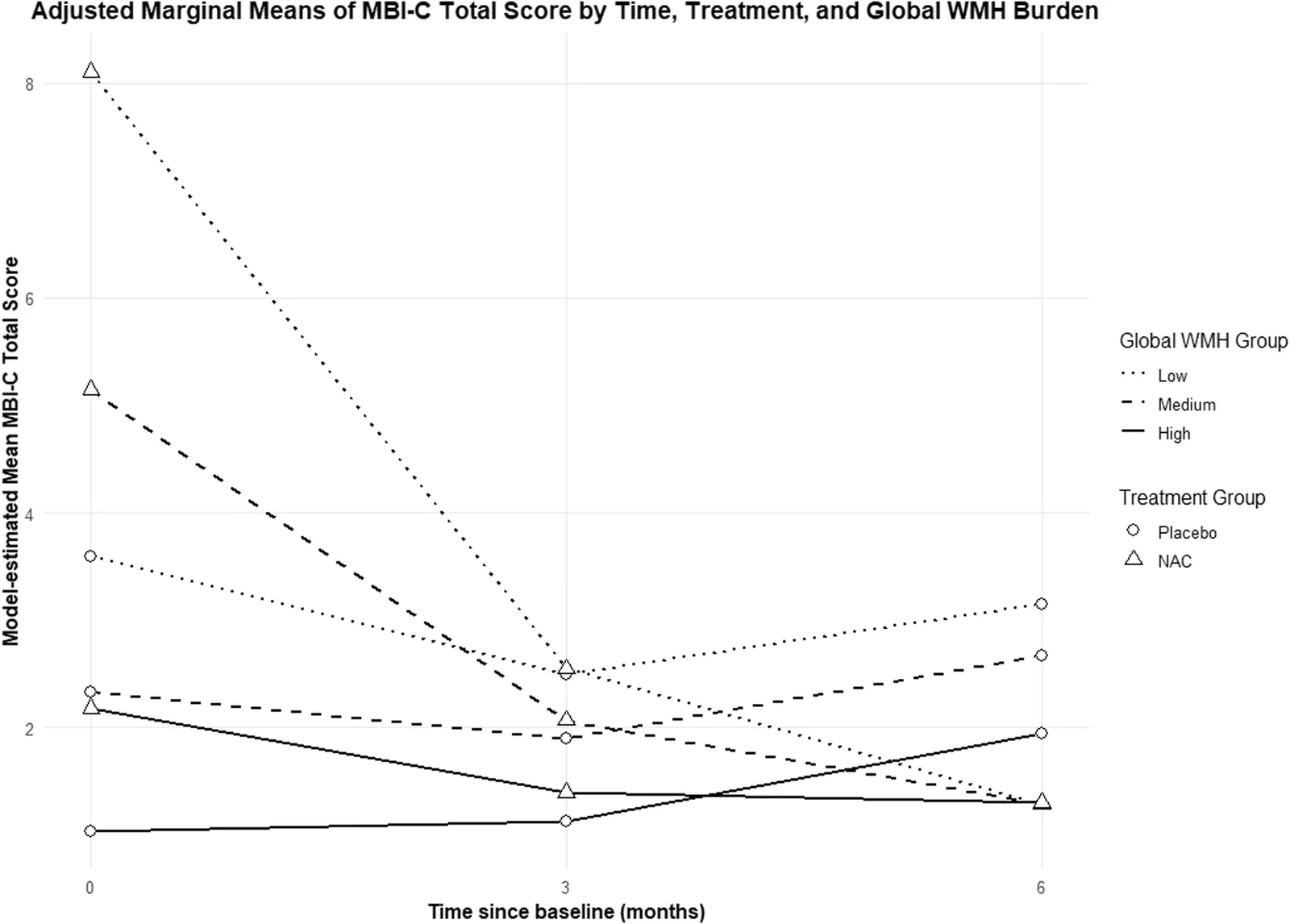
Adjusted marginal means of MBI-C total score over time by treatment assignment (NAC vs placebo) and global WMH tertile. Values represent model-estimated marginal means from the negative-binomial mixed model, adjusted for age, sex, baseline depression, baseline MoCA, vascular risk factors, and preserved brain parenchyma. WMH tertiles are shown for visualization only. Lower scores indicate fewer NPS. Line type denotes WMH tertile (dotted = low WMH; dashed = mid WMH; solid = high WMH).

**Table 4.** Global WMH × time × treatment interaction effects at endpoint on MBI-C total and domain scores.

Outcome	*β* (SE)	Nominal *p*	FDR-adjusted *p*	
MBI-C Total Score	0.258 (0.080)	0.001	0.007	**
Apathy	0.207 (0.089)	0.020	0.049	*
Affective dysregulation	0.132 (0.073)	0.072	0.090	.
Impulse Dyscontrol	0.172 (0.104)	0.099	0.110	
Social Inappropriateness	0.529 (0.294)	0.070	0.090	.

*p < 0.05; **p < 0.01; . *p* < 0.1. MBI-C: Mild Behavioral Impairment Checklist.

For individual domains, the interaction effect was also significant for the apathy domain (*β* = 0.207, SE = 0.089, FDR-adjusted *p* = 0.049) (Table 4). This indicates that participants with lower global WMH burden experienced greater reductions in apathy over 6 months with NAC plus exercise compared to placebo plus exercise (Figure 4). Again, the interaction was not significant for affective dysregulation (FDR-adjusted *p* = 0.090), impulse dyscontrol (FDR-adjusted *p* = 0.110), and social inappropriateness (FDR-adjusted *p* = 0.090), indicating possible trends which did not remain significant after multiple comparison adjustment (Table 4). Figure 5 provides representative baseline T2-FLAIR images from a responder and a non-responder, illustrating the overall supratentorial distribution of WMH and the more pronounced frontal WMH burden in the non-responder, alongside their divergent changes in MBI-C total and apathy domain score between baseline and 6 months.

**Figure 4.**
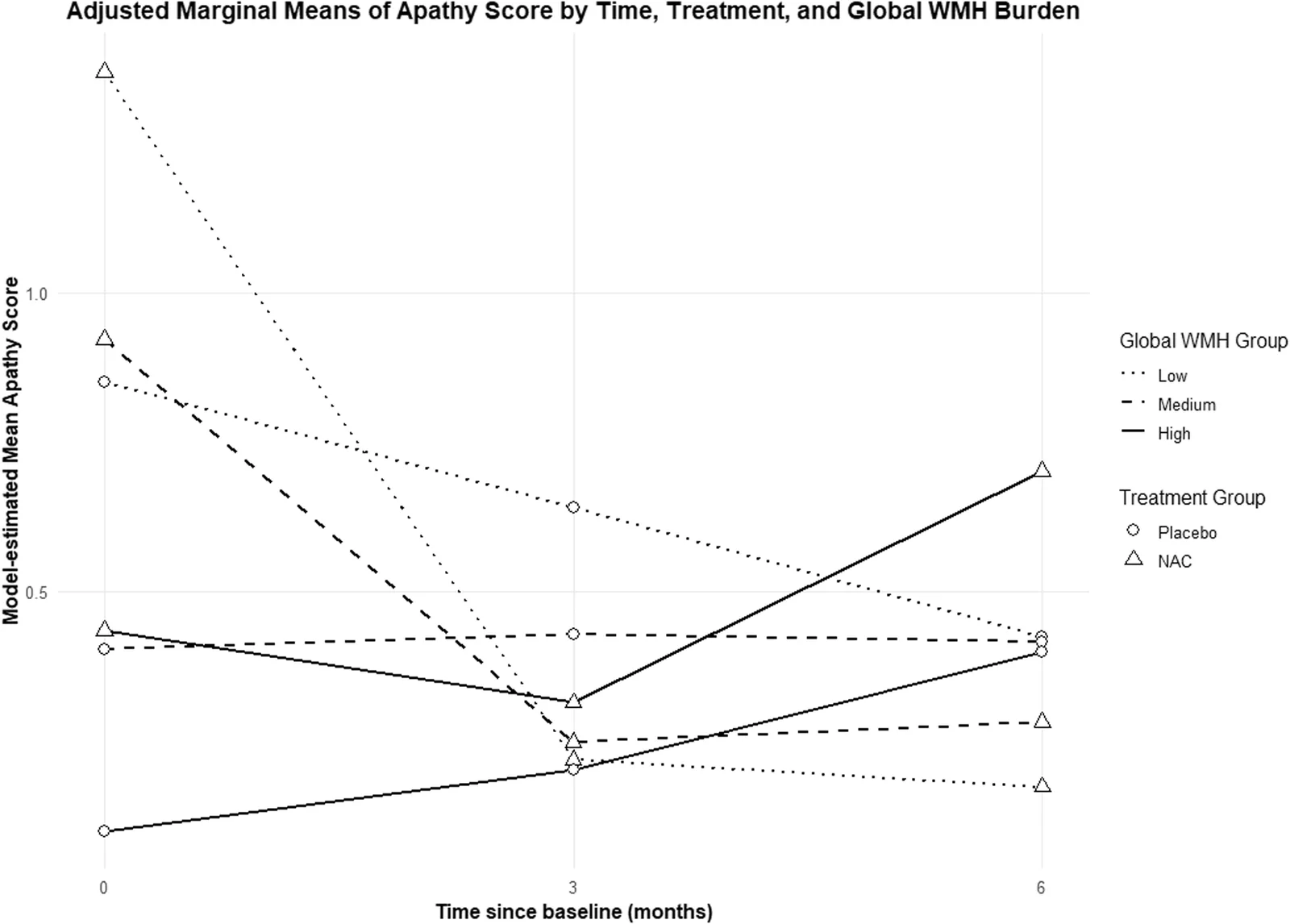
Adjusted marginal means of MBI-C apathy (domain 1) score over time by treatment assignment (NAC vs placebo) and global WMH tertile. Values represent model-estimated marginal means from the negative-binomial mixed model, adjusted for age, sex, baseline depression, baseline MoCA, vascular risk factors, and preserved brain parenchyma. WMH tertiles are shown for visualization only. Lower scores indicate fewer apathy symptoms. Line type denotes WMH tertile (dotted = low WMH; dashed = mid WMH; solid = high WMH).

**Figure 5.**
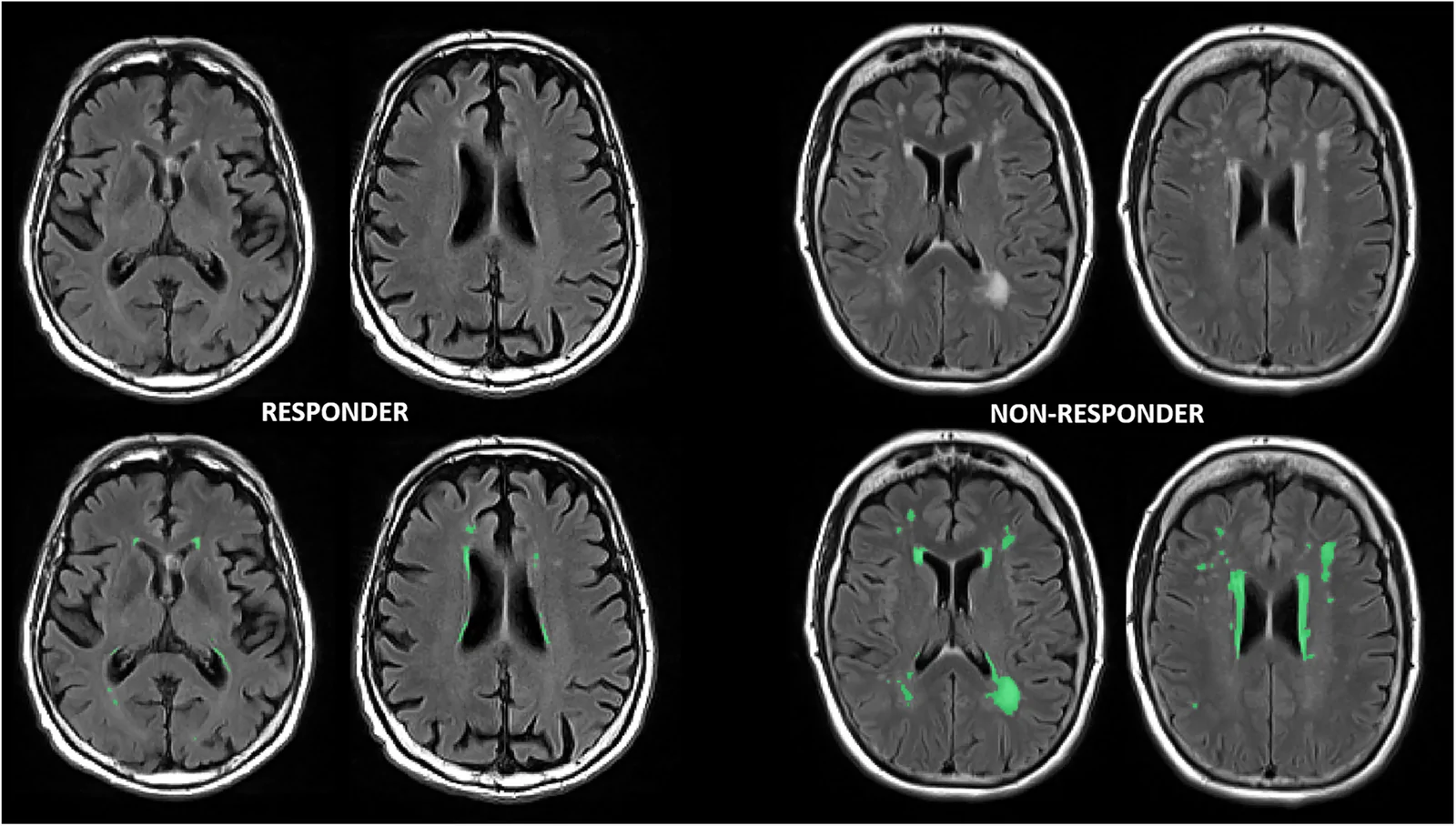
White matter hyperintensity (WMH) burden in a responder and non-responder. The top row shows an axial view of T2-FLAIR MRI and the bottom row shows the same MRI slices with WMH segmentations overlaid in green. For each participant, a mid-level axial slice is shown on the left to depict the overall distribution of supratentorial WMH burden, and a more superior slice emphasizing frontal WMH involvement is shown on the right. The responder, from the NAC group, had relatively low WMH volume and demonstrated a 7-point decrease in MBI-C total score and 5-point decrease in apathy domain score between baseline and endpoint (6 months). The non-responder, from the placebo group, exhibited a high WMH volume and showed an 8-point increase in MBI total score and 6-point increase in apathy domain score over the same interval.

### Exploratory models

Baseline frontal and global WMH burden were highly correlated (Spearman's *ρ* = 0.918, *p* < 0.001), indicating that the two measures were strongly associated. Results from exploratory analyses of regional specificity using baseline occipital, parietal, and temporal WMH burden are shown in Tables 5–7. Significant endpoint time × treatment × WMH interactions were observed for MBI-C total score using both parietal WMH (*β* = 0.192, SE = 0.065, FDR-adjusted *p* = 0.017) and temporal WMH (*β* = 0.176, SE = 0.062, FDR-adjusted *p* = 0.024). These findings indicate that participants with lower baseline parietal and temporal WMH burden experienced greater reductions in overall neuropsychiatric symptoms following NAC plus exercise compared with placebo plus exercise. No significant interactions were observed for occipital WMH after FDR correction. Likewise, parietal and temporal WMH were not significantly associated with treatment-related improvements in apathy, affective dysregulation, impulse dyscontrol, or social inappropriateness following FDR correction (all FDR-adjusted *p* > 0.05).

**Table 5.** Occipital WMH × time × treatment interaction effects at endpoint on MBI-C total and domain scores.

Outcome	*β* (SE)	Nominal *p*	FDR-adjusted *p*	
MBI-C Total Score	0.138 (0.055)	0.012	0.058	.
Apathy	0.104 (0.068)	0.128	0.213	
Affective Dysregulation	0.159 (0.075)	0.033	0.083	.
Impulse Dyscontrol	0.090 (0.068)	0.185	0.232	
Social Inappropriateness	0.166 (0.198)	0.400	0.400	

*. p* < 0.1. MBI-C: Mild Behavioral Impairment Checklist.

**Table 6.** Parietal WMH × time × treatment interaction effects at endpoint on MBI-C total and domain scores.

Outcome	*β* (SE)	Nominal *p*	FDR-adjusted *p*	
MBI-C Total Score	0.192 (0.065)	0.003	0.017	*
Apathy	0.143 (0.071)	0.045	0.101	
Affective Dysregulation	0.097 (0.061)	0.112	0.117	
Impulse Dyscontrol	0.132 (0.084)	0.117	0.117	
Social Inappropriateness	0.457 (0.244)	0.061	0.101	

* *p* < 0.05. MBI-C: Mild Behavioral Impairment Checklist.

**Table 7.** Temporal WMH × time × treatment interaction effects at endpoint on MBI-C total and domain scores.

Outcome	*β* (SE)	Nominal *p*	FDR-adjusted *p*	
MBI-C Total Score	0.176 (0.062)	0.005	0.024	*
Apathy	0.133 (0.070)	0.058	0.141	
Affective Dysregulation	0.084 (0.056)	0.134	0.141	
Impulse Dyscontrol	0.114 (0.078)	0.141	0.141	
Social Inappropriateness	0.336 (0.215)	0.118	0.141	

* *p* < 0.05. MBI-C: Mild Behavioral Impairment Checklist.

## Discussion

This secondary analysis of the MOVE-IT trial provides novel evidence that baseline WMH burden moderates the neuropsychiatric benefits of NAC plus exercise in individuals with vaMCI. Our key findings demonstrate that participants with lower baseline WMH burden, both frontal and global, experienced significantly greater reductions in NPS over six months when treated with NAC plus exercise compared to placebo plus exercise. Importantly, this effect was primarily driven by improvements in apathy, which remained statistically significant after correction for multiple comparisons.

The significant three-way interactions between treatment, time and both frontal and global WMH burden on overall NPS support our hypothesis and align with established theories of vascular cognitive impairment. These findings are particularly relevant given that apathy has emerged as one of the most prominent and clinically significant NPS in vaMCI, associated with accelerated cognitive decline and increased dementia risk.^3,8,10^ The consistency of results across both frontal and global WMH measures strengthens confidence in our findings and suggests that overall cerebrovascular burden and location-specific damage, may both be the critical factors determining treatment responsiveness. Importantly, these associations were significant despite adjustment for regional and global brain parenchymal fraction, suggesting that the moderating effect of WMH burden was not solely attributable to concomitant brain atrophy. This finding indicates that WMH burden provides prognostic information beyond preserved brain parenchyma. Exploratory analyses examining other lobar WMH distributions further refined these findings. While lower parietal and temporal WMH burden also predicted greater reductions in overall MBI-C score, neither region was associated with improvements in apathy following FDR correction. These findings suggest that treatment responsiveness for overall neuropsychiatric symptom burden may reflect more widespread cerebrovascular pathology, whereas the association with apathy appears more closely linked to frontal-subcortical network integrity.

Given the strong correlation between frontal and global WMH burden, the frontal and global analyses should not be interpreted as independent tests of separate pathological processes. Rather, the similar findings across both measures suggest that the observed treatment moderation effect was robust to whether cerebrovascular burden was quantified using a frontal lobe-specific or global WMH measure. The exploratory regional analyses further demonstrated that frontal, but not occipital, temporal, or parietal, WMH significantly predicted improvement in apathy, supporting some degree of regional specificity despite the strong correlation between frontal and global WMH burden.

The observed pattern whereby individuals with lower WMH burden show greater treatment benefits likely reflects the underlying pathophysiology of white matter damage and its impact on therapeutic mechanisms. Frontal WMHs represent chronic ischemic damage to white matter tracts, particularly those connecting frontal cortical regions with subcortical structures essential for motivation and goal-directed behavior.^44^ However, cerebrovascular injury is often widespread and can disrupt fronto-subcortical and fronto-parietal networks that support motivation and affect regulation.^45^ Greater global WMH burden may contribute to neuropsychiatric symptoms via network disconnection and secondary grey matter atrophy in frontal and posterior regions, consistent with apathy-related cortical thinning and volume loss in frontal and temporo-parietal regions reported in mild behavioral impairment.^46^ Low levels of endogenous antioxidants, especially GSH, have been associated with greater WMH volume and vascular brain injury, supporting a link between impaired redox defense, white matter damage, and small-vessel pathology.^47,48^ NAC is a precursor of GSH and increases brain and vascular GSH availability, thereby protecting small vessels and white matter from oxidative injury.^49,50^ From this perspective, individuals with higher WMH burden and greater oxidative stress may have a greater need for antioxidant support. However, extensive WMH burden may also reflect more advanced microvascular injury, chronic axonal disruption, and reduced cellular repair capacity, thereby limiting the potential for recovery despite treatment. Conversely, individuals with lower WMH burden may retain sufficient structural integrity and neuroplastic potential to benefit from restored GSH-mediated antioxidant defenses and the combined effects of NAC and exercise.^3,8,51^

The predominant effect on apathy, rather than other neuropsychiatric domains, aligns with the vascular apathy hypothesis, which posits that cerebrovascular disease can be a primary cause of motivational deficits.^14^ NAC's antioxidant properties may help mitigate ongoing oxidative damage to these vulnerable circuits,^52,53^ while structured exercise provides complementary benefits through improved cerebrovascular function, enhanced neuroplasticity, and direct effects on motivation and mood.^54–56^ The synergistic combination of these interventions appears most effective in individuals with less severe underlying vascular pathology. Across major neurocognitive disorders, higher frontal and global WMH burden is consistently associated with greater apathy severity.^14^ However, in this 6-month trial, the largest apathy reductions were observed in participants with lower baseline WMH burden, again suggesting that substantial structural reserve may be required and that any potential benefits among those with higher WMH burden may emerge over longer treatment durations than were captured here.

### Limitations

Several limitations should be considered when interpreting these results. First, the sample size was relatively small (*n* = 51), which may limit the generalizability of findings and the power to detect smaller effect sizes in individual neuropsychiatric domains. The highly educated, predominantly male, White sample with low MBI scores, recruited from a CR program may not be representative of the broader vaMCI population, potentially limiting external validity. The study design precluded examination of NAC or exercise effects in isolation, as both treatment arms included exercise training. While this design reflects real-world CR practices, it prevents determination of whether the observed moderation effects are specific to NAC, exercise, or their combination. Additionally, the 24-week follow-up period, while sufficient to detect acute intervention effects, does not address longer-term sustainability of neuropsychiatric improvements or potential delayed treatment responses. Because apathy severity is variable at baseline, some changes over time may reflect regression to the mean (RTM) rather than true group differences. All inferential conclusions were therefore based on mixed-effects models with random intercepts, which account for stable between-person differences and focus inference on within-person change over time. Although RTM may contribute to changes in individual symptom scores over time, it would not be expected to systematically generate the observed time × treatment × WMH interactions reflecting differential treatment responses. As such, this approach successfully isolates the true treatment effect from RTM artifacts. Additionally, while we controlled for multiple comparisons using FDR correction, the exploratory nature of domain-specific analyses and the multiple statistical models tested warrant cautious interpretation of findings. Finally, MRI-based WMH quantification was limited to baseline only. Evaluation of longitudinal WMH data will help clarify this relationship. Future studies with larger, more diverse samples and longer follow-up periods are needed to validate these preliminary findings and establish the importance of WMH burden in predicting response in vaMCI populations.

### Conclusion

This study provides the evidence that baseline WMH burden serves as a significant moderator of neuropsychiatric treatment response in vaMCI. Our findings demonstrate that individuals with lower WMH burden, both frontal and global, experience substantially greater reductions in NPS, particularly apathy, following six months of NAC plus exercise compared to placebo plus exercise. The consistency of findings across different WMH measures strengthens confidence in these results. These results highlight the potential for personalized intervention approaches taking into consideration underlying cerebrovascular pathology. Ultimately, these findings advance our understanding of the complex interplay between vascular pathology, NPS, and therapeutic responsiveness, and support the importance of optimizing care for cerebrovascular risk factors in this vulnerable population.

## Supplemental Material

sj-docx-1-alz-10.1177_13872877261490542 - Supplemental material for White matter hyperintensities moderate neuropsychiatric response to N-acetylcysteine and exercise therapy in vascular mild cognitive impairment Supplemental material, sj-docx-1-alz-10.1177_13872877261490542 for White matter hyperintensities moderate neuropsychiatric response to N-acetylcysteine and exercise therapy in vascular mild cognitive impairment by Ethan Mah, Damien Gallagher, Yejin Kang, Fuqiang Gao, Joel Ramirez, Zahinoor Ismail, Sandra E. Black, Xingshan Cao, Nathan Herrmann, Susan Marzolini, Paul Oh, Jinghan Jenny Chen, Kate Survilla, Walter Swardfager, Danielle Vieira, Mark J. Rapoport and Krista L. Lanctôt in Journal of Alzheimer's Disease

## Data Availability

The data supporting the findings of this study are available on request from the corresponding author. The data are not publicly available due to privacy or ethical restrictions.
