# Supplementary material for: White matter hyperintensities moderate neuropsychiatric response to N-acetylcysteine and exercise therapy in vascular mild cognitive impairment
Source: J Alzheimers Dis. 2026 Oct 7;114(2):874–87. doi: 10.1177/13872877261490542 (PMC13740187; doi:10.1177/13872877261490542)
Supplement: Supplementary file 1 [file sj-docx-1-alz-10.1177_13872877261490542.docx]

**Supplemental Material**

**White matter hyperintensities moderate neuropsychiatric response to N-acetylcysteine and exercise therapy in vascular mild cognitive impairment**

**Supplemental Figure 1.** Observed individual participant trajectories for MBI-C total scores from baseline to 3 and 6 months, stratified by baseline frontal WMH burden (low versus high, defined by a median split) and treatment group. Each line represents an individual participant. These raw data are presented for visualization only and do not reflect the adjusted mixed-effects model estimates reported in the primary analyses.

**Supplemental Figure 2.** Observed individual participant trajectories for MBI-C apathy scores from baseline to 3 and 6 months, stratified by baseline frontal WMH burden (low vesus high, defined by a median split) and treatment group. Each line represents an individual participant. These raw data are presented for visualization only and do not reflect the adjusted mixed-effects model estimates reported in the primary analyses.
